# New tools to screen wild peanut species for aflatoxin accumulation and genetic fingerprinting

**DOI:** 10.1186/s12870-018-1355-9

**Published:** 2018-08-15

**Authors:** Renee S. Arias, Victor S. Sobolev, Alicia N. Massa, Valerie A. Orner, Travis E. Walk, Linda L. Ballard, Sheron A. Simpson, Naveen Puppala, Brian E. Scheffler, Francisco de Blas, Guillermo J. Seijo

**Affiliations:** 1 USDA-ARS-NPRL, National Peanut Research Laboratory (NPRL), 1011 Forrester Dr. S.E, Dawson, GA 39842 USA; 2 USDA-ARS-GBRU, Genomics and Bioinformatics Research Unit, 141 Experiment Station rd, Stoneville, MS 38776 USA; 3 New Mexico State University, Agricultural Science Center at Clovis, 2346 SR 288, Clovis, NM 88101 USA; 4 Facultad de Ciencias Exactas y Naturales y Agrimensura, Universidad Nacional del Nordeste, Av. Libertad 5470, C.P, 3400 Corrientes, Argentina; 5 Instituto de Botánica del Nordeste, (UNNE-CONICET), Casilla de Correo 209, 3400 Corrientes, Argentina

**Keywords:** Fingerprinting, groundnut, peanut, molecular markers, aflatoxin, *Arachis*, *Aspergillus flavus*

## Abstract

**Background:**

Aflatoxin contamination in peanut seeds is still a serious problem for the industry and human health. No stable aflatoxin resistant cultivars have yet been produced, and given the narrow genetic background of cultivated peanuts, wild species became an important source of genetic diversity. Wild peanut seeds, however, are not abundant, thus, an effective method of screening for aflatoxin accumulation using minimal seeds is highly desirable. In addition, keeping record of genetic fingerprinting of each accession would be very useful for breeding programs and for the identification of accessions within germplasm collections.

**Results:**

In this study, we report a method of screening for aflatoxin accumulation that is applicable to the small-size seeds of wild peanuts, increases the reliability by testing seed viability, and records the genetic fingerprinting of the samples. Aflatoxin levels observed among 20 wild peanut species varied from zero to 19000 ng.g^-1^ and 155 ng.g^-1^ of aflatoxin B_1_ and B_2_, respectively. We report the screening of 373 molecular markers, including 288 novel SSRs, tested on 20 wild peanut species. Multivariate analysis by Neighbor-Joining, Principal Component Analysis and 3D-Principal Coordinate Analysis using 134 (36 %) transferable markers, in general grouped the samples according to their reported genomes. The best 88 markers, those with high fluorescence, good scorability and transferability, are reported with BLAST results. High quality markers (total 98) that discriminated genomes are reported. A high quality marker with UPIC score 16 (16 out of 20 species discriminated) had significant hits on BLAST2GO to a pentatricopeptide-repeat protein, another marker with score 5 had hits on UDP-D-apiose synthase, and a third one with score 12 had BLASTn hits on La-RP 1B protein. Together, these three markers discriminated all 20 species tested.

**Conclusions:**

This study provides a reliable method to screen wild species of peanut for aflatoxin resistance using minimal seeds. In addition we report 288 new SSRs for peanut, and a cost-effective combination of markers sufficient to discriminate all 20 species tested. These tools can be used for the systematic search of aflatoxin resistant germplasm keeping record of the genetic fingerprinting of the accessions tested for breeding purpose.

**Electronic supplementary material:**

The online version of this article (10.1186/s12870-018-1355-9) contains supplementary material, which is available to authorized users.

## Background

Presence of aflatoxins in peanut meal caused massive mortality of poultry almost 60 years ago [1]. Since then, significant effort was made to obtain peanut varieties with resistance to the accumulation of aflatoxins [2–6]; and, though progress has been made, there are no cultivated peanuts with stable resistance to these mycotoxins [6]. Aflatoxins are secondary metabolites produced mainly by the fungi *Aspergillus flavus* Link ex Fries and *Aspergillus parasiticus* Speare; are highly carcinogenic compounds [7] and accumulate in seeds of many crops. These polyketide-derived mycotoxins cause acute hepatotoxicity and immunosuppression [8, 9] as well as human and animal deaths by aflatoxicosis (Azziz-Baumgartner et al. 2005). In the United States, aflatoxins cost farmers and the peanut industry millions in losses each year [10, 11], whereas losses due to aflatoxin contamination of peanut exports worldwide account for as much as $450 million [12].

Cultivated peanuts have a narrow genetic background [13], due to multiple factors, *e.g*., biology, habitat, origin, ploidy, section, as summarized by Mallikarjuna et al. (2011) [14]. This is evident when using molecular markers, which normally show low levels of polymorphism [15–17] in peanut. Thus, for decades, researchers have recognized the need to broaden the genetic background of peanut by incorporating wild species germplasm [18–20], and wild relatives of the cultigen *A. hypogaea* L. have been recognized as an important source of genes for resistance to biotic and abiotic stresses [21]. The peanut collection at USDA National Plant Germplasm System consists of 9,321 accessions of cultivated peanut (*Arachis hypogaea*) and 655 accessions from 66 wild *Arachis* species; most accessions (44%) were collected from South America [22] in a multinational effort that involved many expeditions to the center of origin [19, 20, 23]. The peanut germplasm collection in Griffin is composed of accessions collected from 102 countries.

The genetics of aflatoxin resistance is complex, involving pre-harvest response, post-harvest, genotype x environment interaction, and host-pathogen interactions [24]; however, the use of *in vitro* seed colonization (IVSC) provides useful information and has resulted in six new sources of resistance being identified and confirmed by other researchers [24]. The IVSC method was used to identify two interesting *Arachis* species with low accumulation of aflatoxin, *A. cardenasii* Krapovickas and Gregory and *A. duranensis* Krapovickas & Gregory, and they were targeted for introgression into *A. hypogaea* L. [6]. Since wild peanut seeds are not readily available, screenings for aflatoxin resistance trait should use minimal seeds. Given the large variability of aflatoxin accumulation in seeds, even under controlled conditions, experiments usually require hundred grams of seeds or more and large planting areas [25, 26]. Though screening with much smaller amounts, 5 g of seed per replicate have been reported [6].

No general screening of phenotypic and genetic fingerprinting characteristics has been done of the germplasm collection of wild peanut species, particularly in relation to aflatoxin accumulation. At the NPRL we have developed a method to screen for aflatoxin accumulation by inoculating few seeds with an aflatoxigenic *Aspergillus* and analyzing each half cotyledon using ultra performance liquid chromatography (UPLC) [27–29]. Here we developed a method adapted to the small size of wild peanut seeds, performing single seed analysis and added reliability to the results by testing the viability of each seed, while keeping record of each accession using novel SSR markers. We provide a final useful set of tools not only for the cost-effective screening of the germplasm collection, but also generating a genotypic and phenotypic database that can be used by peanut breeders.

## Methods

### Microsatellite development

DNA of *Arachis hypogaea* subsp. *fastigiata* var. *fastigiata* cv. New Mexico Valencia C peanut seeds (provided by Dr. Naveen Puppala) was extracted using DNeasy Plant Maxi Kit (Qiagen, Valencia, CA) and SSR-enriched libraries were generated [30]. Briefly, DNA was digested with restriction enzymes *Alu*I, *Hae*III, *Dra*I, and *Rsa*I (New England Biolabs, Ipswich, MA), then the blunt-end DNA fragments were A-tailed with *Taq*-DNA Polymerase (Promega, Madison, WI) and ligated to a linker made from oligos SSRLIBF3: 5’- CGGGAGAGCAAGGAAGGAGT-3’ and SSRLIBR3 5’Phos-CTCCTTCCTTGCTCTCTCCCGAAAA-3’ [30]. Ligated fragments were amplified by PCR, and the product was hybridized to groups 2 and 3 of biotinylated oligo repeats [31]. Hybridizations were followed by an extension step of 10 min at 68°C as indicated in Hayden et al. [32] in the presence of High Fidelity *Taq* Polymerase (Invitrogen, Carlsbad, CA). Sequences containing repeats were captured using streptavidin-coated magnetic beads M-270 (Invitrogen, Carlsbad, CA) in a Labquake tube shaker/rotator (Barnstead/Thermoline, Dubuque, IA) at 22°C for 1 h [33]. Elution of the DNA from the biotinylated oligos was done with 60 μl MilliQ water and the eluate was PCR amplified for 10 cycles as indicated for the ligation step (Techen et al., 2010). PCR products were sequenced by pyrosequencing in a Roche 454-GS Junior (Roche, Indianapolis, IN). Sequences were assembled with Roche 454 gsAssembler version 2.0 (Roche, Branford, CT). Repeats were searched using SSRFinder [34] and primers were designed using Primer3 [35] for a melting temperature of 63 ± 1°C, and 5 base pairs (bp) as maximum overlapping with repeats. DNA sequences containing repeats were BLAST to the genomes of *Arachis ipaënsis* Krapovickas & Gregory and *Arachis duranensis* Krapovickas & Gregory [36] and to gene ontology (BLAST2GO®) [37]. Results of BLAST to *A. duranensis* and *A. ipaënsis* are shown in Additional file 1: Table S1, and results of BLAST2GO are listed in Additional file 2: Table S2.

### Fingerprinting of 20 wild species of peanuts from the germplasm collection

The first 20 wild species of peanuts alphabetically listed in the bank of germplasm were chosen for the experiments, seeds of one accession per species, Table 1, were obtained from the Plant Genetic Resources Conservation Unit (PGRCU), Griffin, GA. Twenty was considered a manageable number of accessions to develop a screening method, though the goal is to screen the rest of the collection. Genomic DNA was extracted from seeds of 20 wild peanut species and the control cultivar Georgia-11J [38], using DNeasy Plant Mini Kit (Qiagen, Valencia, CA). A total of 288 newly developed peanut SSRs (Additional file 1: Table S1), in addition to 92 Insertion/Deletion (InDel) markers [39] and microsatellites reported in the literature [21, 40–44](Additional file 2: Table S2), formed a list of 373 markers used for fingerprinting. Forward primers were 5’ tailed with the sequence 5’-CAGTTTTCCCAGTCACGAC-3’ [45] and reverse primers were tailed at the 5’ end with the sequence 5’-GTTT-3’ to promote non-template adenylation [46]. Primer 5’-CAGTTTTCCCAGTCACGAC-3’ labeled with 6-carboxy-X-rhodamine (ROX) (IDT-Technologies, Coralville, IA) was used for amplification of 10-ng DNA using Titanium *Taq* DNA Polymerase (Clontech, Mountain View, CA) as reported before [47]. Fluorescently-labeled PCR fragments were analyzed by capillary electrophoresis on an ABI 3730XL DNA Analyzer and data processed using GeneMapper 4.0 (both from Applied Biosystems, Foster City, CA). Presence of alleles was converted to a binary matrix and Principal Component Analysis (PCA) was performed to identify patterns of genetic relationships using the R package adegenet version 2.0.1 [48] in R version 3.4.0 [49]. Cluster analysis by Neighbor Joining (NJ) and 3D-Principal Coordinate Analysis (3D-PCoA) were calculated using NTSYSpc v. 2.2 (Exeter Software, Setauket, NY). Confidence level of the generated dendrogram was assessed by bootstrap with 5000 re-sampling [50, 51]. Based on transferability, polymorphism and ease to score, data from the best markers were used to calculate their actual discriminating power by running UPIC scripts [52], and to find the most informative combination of markers that can distinguish all the samples tested. UPIC scripts were also used to calculate the heterozygosity of the samples at each of the loci.

**Table 1:** Arachis genotypes included in this study

Section and Species	2n	Genome	PI number	Life cycle	Source
Arachis					
Arachis batizocoi Krapov. & W.C. Gregory	20	K	PI 298639	Perennial or biennial	Bolivia
Arachis benensis Krapov., W.C. Gregory & C.E. Simpson	20	F	PI 475877	Annual	Bolivia
Arachis cardenasii Krapov. & W.C. Gregory	20	A	PI 475994	Perennial	Bolivia
Arachis correntina (Burkart) Krapov. & W.C. Gregory	20	A	PI 210554	Perennial	- -
Arachis cruziana Krapov., W.C. Gregory & C.E. Simpson	20	K	PI 476003	Annual	Bolivia
Arachis decora Krapov., W.C. Gregory & Valls	18	Unknown	PI 666082	Annual	Brazil
Arachis diogoi Hoehne	20	A	PI 276235	Perennial	Paraguay
Arachis duranensis Krapov. & W.C. Gregory	20	A	PI 219823	Annual	Argentina
Arachis glandulifera Stalker	20	D	PI 468343	Annual	Bolivia
Arachis helodes Martius ex Krapov. & Rigoni	20	A	PI 468144	Perennial	Brazil
Arachis ipaënsis Krapov., W.C. Gregory	20	B	PI 468322	Annual	Bolivia
Arachis magna Krapov. et al.	20	B	PI 598184	Annual	- -
Arachis monticola Krapov. & Rigoni	40	AB	PI 468196	Annual	Argentina
Erectoides					
Arachis benthamii Handro	20	E	PI 468162	Perennial	Brazil
Arachis cryptopotamica Krapov. & W.C. Gregory	20	E	PI 468165	Perennial	Brazil
Arachis hermannii Krapov. & W.C. Gregory	20	E	PI 604847	Perennial	Brazil
Heteranthae					
Arachis dardani Krapov. & W.C. Gregory	20	H	PI 591364	Annual or biennial	Brazil
Procumbentes					
Arachis chiquitana Krapov., W.C. Gregory & C.E. Simpson	20	PR	PI 476006	Perennial	Bolivia
Rhizomatosae					
Arachis burkartii Handro	20	R1	PI 468162	Perennial	Brazil
Arachis glabrata var. hagenbeckii Benth. (Harms ex. Kuntze) F.J. Herm.	40	R2	PI 262839	Perennial	Paraguay

### Screening of wild peanut species for aflatoxin accumulation and viability

Long term storage and maturity level at harvest can affect the viability of seeds within the germplasm collection, therefore, each seed tested for aflatoxin accumulation was also screened for viability through germination under *in vitro* culture conditions. Four peanut seeds of each of the 20 wild species of peanut, Table 1, were challenged with *Aspergillus flavus* (Link) NRRL3357 to quantify aflatoxin accumulation using a previously reported method [27] with some modifications due to the small size of wild peanut seeds. Seeds of peanut cultivar Georgia-11J [38] were used as controls and processed as the wild species. The introduced changes were: a) the use of 1 % sodium hypochlorite solution for disinfection (instead of 2 %); b) 3 h imbibition (instead of 2 h); c) seeds with testa (skins) were cut in two pieces (cross section) without separating the cotyledons, d) use of only the apical (distal) half for inoculation, e) the inoculum size per half seed was 0.2 μL of a freshly made spore suspension in water (3 x 10^5^ spores/mL) of a 5-7 day-old culture of *A. flavus* NRRL 3357 grown on potato-dextrose agar (PDA). Water-agar Petri dishes containing the inoculated half seeds were incubated in the dark at 29 ± 1 °C; after 72 h incubation each half seed was placed in separate pre-weighed 4 mL vials and weighed; the weights were recorded, and the seeds were stored at -80°C until chemical analysis (no longer than 3 weeks). To assess the viability of the seeds, the proximal half (containing the embryonic axis) was placed in test tubes (25 x 250 mm) containing 18 mL of Hoagland’s No. 2 (0.4 g/L) (H2395, Millipore-Sigma) and agar (6 g/L). Seeds in Hoagland’s medium were incubated at 28 ± 1°C in the dark for seven days, and 16 h light/8 h dark thereafter. Four seeds per peanut accession were tested for aflatoxin accumulation and viability assessment as germination. Mycelium growth after inoculation, and seed germination were documented by photographs.

### Aflatoxin analysis

Seed halves inoculated with *A. flavus* were transferred from the 4 mL vials to 2 mL reinforced tubes containing six 2.8 mm ceramic beads (cat #: 19-649 & 19-646-3, respectively, Omni International, Kennesaw, GA) and 0.5 mL methanol; the samples were pulverized for 20 sec at 5.5 m/s in a Bead Ruptor 24 homogenizer (Omni International). Using a Pasteur pipette, the liquid was transferred to another Pasteur pipette previously fitted with a glass fiber plug and 70 mg Celite ®545 (Sigma); the liquid was forced through the pipette column using nitrogen gas and collected into a 700 μL Ultra-High Performance Liquid Chomatographer (UPLC) auto sampler vial (part # 186005221, Waters, Milford, MA). Samples were subjected to aflatoxin analysis using a Waters Acquity UPLC instrument equipped with a matching UPLC H-class Quaternary Solvent Manager, UPLC Sample Manager, UPLC Fluorescent Detector (FLR), and an Acquity UPLC BEH C18 2.1 mm x 50 mm, 1.7 μm column. Water (A), methanol (B), and acetonitrile (C) were used in the following gradient: initial conditions, 64 % A/ 23 % B/ 13 % C, held for 4 min, changed to 6 % A/ 40 % B/ 54 % C in 0.01 min, held for 4 min, changed to initial conditions in 0.01 min, held isocratic for 2 min before next injection. The flow rate was 0.3 mL/min. The column was maintained at 40 ^o^C in the system column heater. Concentrations of aflatoxins were determined by reference to peak areas of corresponding commercial standards (calibration curve). The lowest detection limit for aflatoxin B_1_ was 0.10 ng.g^-1^ and 0.01 ng.g^-1^ for aflatoxin B_2_. Statistical tests were performed using the statistical package Sigma Plot v. 12.5 (Systat Software Inc., San Jose, CA).

## Results

### Sequencing, SSR isolation and primer design

**Fig. 1:**
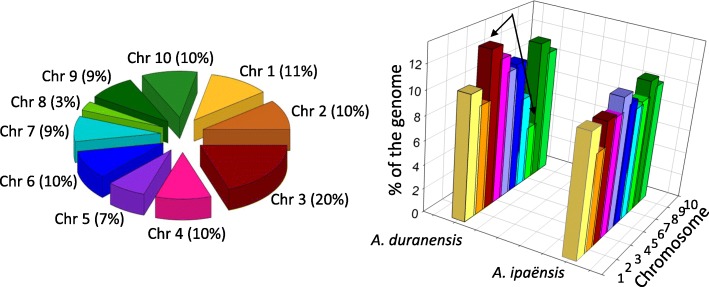
Left: Percentage of markers from this study, found in the same corresponding chromosomes of *A. ipaënsis* and *A. duranensis* (total 193 markers), and their distribution by chromosome. Right: Size of each of the 10 chromosomes of *A. ipaënsis* and *A. duranensis*, in proportion to their entire genomes. Arrows indicate the larger size of chromosome three and smaller size of chromosome eight in *A*. *duranensis*. Chr: chromosome

High throughput sequencing and assembly of SSR-enriched libraries generated from Valencia peanut resulted in 2,974 contigs and 34,344 singletons; a total of 11,600 SSRs were detected, and 879 unique primer sets were designed. The first 288 markers in that list, a manageable number of primers (3 plates of 96 wells), were tested and reported in the present work. A total of 265 sequences containing the 288 SSRs were uploaded to the National Center for Biotechnology Information (NCBI) with GenBank with accession number: KY177179 to KY177444, sequence names in the database match the names of the SSR markers reported here. The frequency of repeat motifs in the 288 microsatellites was 58, 161, 61, 3 and 5, for di, tri, tetra, penta, and hexa nucleotide (nt), respectively; whereas the number of repeat units in the microsatellites ranged from 4 to 64 units. Numerous types of repeats were detected; the most abundant repeat motifs were: ATC, AAG, TCG, GA and TTC, though none of them exceeded 9 % of the total number of repeats. Repeat motifs for each primer set, primer sequences, alleles per marker and amplicon sizes are described in Additional file 1: Table S1. Also included in this table are the number of alleles and amplicon sizes for markers obtained using published markers, and the results of BLAST2GO for the sequences. Since the average length of DNA sequences containing markers was 284 bp (Additional file 1**:** Table S1) and the amplicon sizes ranged from 94-489 bp, it is highly likely that marker polymorphisms occurred within the open-reading frames of sequences that had significant hits on BLAST2GO. BLAST results against the genomes of *Arachis ipaënsis* (Krapovickas & Gregory) and *Arachis duranensis* (Krapovickas & Gregory) indicating chromosome location are shown in Additional file 3: Table S3. A total of 373 markers were used to screen 20 wild species of peanut. From these 373 markers, a total of 193 (52%) were located in the same corresponding chromosomes of *A. ipaënsis* and *A. duranensis*. The distribution of these 193 markers showed that all 10 chromosomes were represented by the markers used in this study, with 3 % of the markers on chromosome 8, to 20 % on chromosome 3, Fig. 1. The uneven distribution was apparently related to the small size of chromosome 8 and large size of chromosome 3 in *A. duranensis*, Fig. 1. Included in the fingerprinting analysis, there were 85 markers, both SSRs and InDels, obtained from the literature, [21, 39–43], a list is summarized in Additional file 2: Table S2. Only few markers were detected only in the A or B genomes using BLAST analysis on *A. duranensis* and *A. ipaënsis*, these were NPRL_contig00098a (only A), and NPRL_contig00139a and NPRL_contig02833a (only B).

Out of 373 markers tested, only two (0.5%), [NPRL_contig00139a (this work) and AHBGSI1002D05 [41]], were monomorphic and amplified in all 20 wild peanut species; another 10 markers had single amplicons but presented null alleles in one or more samples, therefore were not considered monomorphic (Additional file 1: Table S1). From the 325 markers that resulted in amplification, the total number of alleles observed was 2473, the number of alleles per marker ranged between 2 and 26 , with an average of 7.7 alleles per locus, data shown in Additional file 1: Table S1. A total of 130 markers tested, either failed to amplify some of the samples, presented multiple amplicons with stutters, or had very low fluorescence in one or more samples; another 109 markers amplified in all the samples but were not easy to score, thus, these 239 (130+109) markers were excluded from the multivariate analysis. The remaining 134 markers (104 from this work, and 30 from the literature) were transferable across all 20 accessions of wild peanuts, had no null alleles, had good levels of fluorescence and were easy to score. These 134 markers were used in cluster analysis by Neighbor-Joining (NJ), as well as in Principal Component Analysis (PCA) and 3D-Principal Coordinate Analysis (3D-PCoA).

**Fig. 2:**
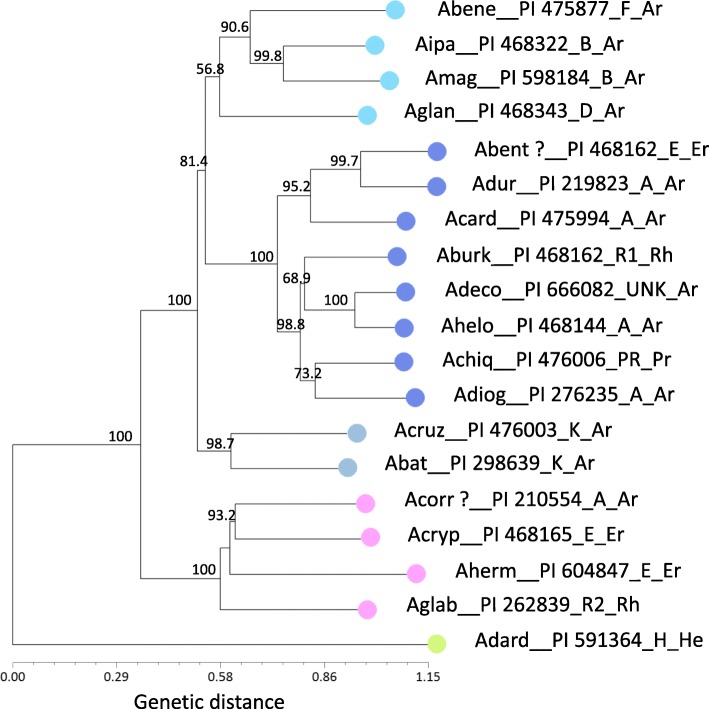
Neighbor-joining dendrogram based on Nei’s standard genetic distance using 134 molecular markers on 20 wild species of peanut. The samples are colored to indicate the overall grouping of species by genome type (dark blue: A, pink: E, grey: K, green: H, light blue: B D F). The same colors were used for each sample in PCA and 3D-PCoA analyses. Only bootstrap values higher than 50 are shown at the nodes. For graphic clarity the species names were shortened, please see List of Abbreviations. Species names are followed by PI, genome type and Section (Ar: Arachis; Er: Erectoides; He: Heteranthae; Pr: Procumbentes; Rh: Rhizomatosae). 98 good-quality markers that discriminated genomes follow the same color code in Additional file 4: Table S4

**Fig. 3:**
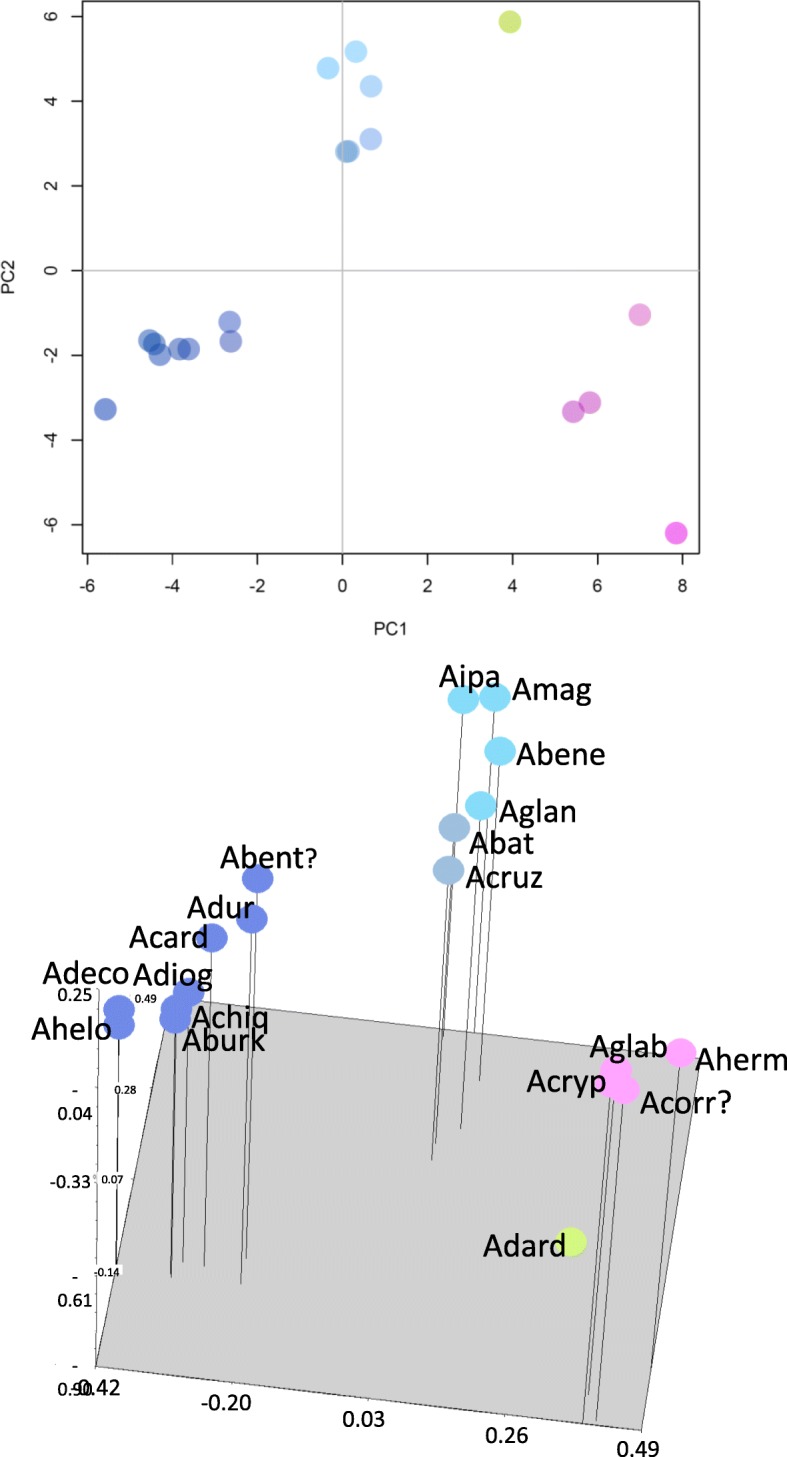
Graph of the first two axes from a principal component analysis (PCA) (Top) and 3-Dimention-Principal Coordinate Analysis (3D-PCoA) (Bottom) of 20 wild species of peanut using 134 molecular markers (including SSRs and InDels). The first component explains 16 % and the second 11 % of the total genetic variation; and the first 3 PCoA dimensions explained 34 %, 25 % and 21 % (total 80%) of the genetic variation. For graphic clarity the species names were shortened, please see List of Abbreviations. Color code as in Fig. 2

Cluster analysis by NJ, separated the wild peanut accessions into five main groups mostly according to their genome types reported in the literature; thus, the group with the dark-blue symbol corresponded in general to A genome, pink symbol grouped mostly E genome, green symbol was represented by the H genome, grey symbol grouped the K genome, and light-blue included B D and F genomes, Fig. 2. Similar results were observed for PCA, the first three components explained 16 %, 11 % and 9 % (total 36 %) of the genetic variation, and data are plotted for components PCA1 and PCA2 in Fig. 3. The results of 3D-PCoA largely corresponded to those obtained through NJ cluster analysis and PCA, though a more clear definition of groups was observed for the first three coordinates, which explained 34%, 25%, and 21% (total 80%) of the genetic variation, Fig. 3. Similar colors were used for the graphics of NJ, PCA and 3D-PCoA to reflect the consistency of grouping patterns obtained by different analyses. The group formed by B D F genome (light blue symbol) was close to *Arachis batizocoi* (Krapovickas & Gregory) and *Arachis cruziana* (Krapovickas, Gregory & Simpson) K genome (grey symbol) both in PCA and PCoA; in NJ the B D F genome group was slightly more distant from the K genome group, though in this case the confidence level for this clade was relatively low, only 56.8%, Fig. 2. A total of 98 high-quality markers that discriminated genomes (*e.g*., H, D, F, AB, E, R1, R2) or groups of genomes (*e.g*., E+R2, B+F, D+H) were organized by their quality (from excellent to doable) and are listed in Additional file 4: Table S4. The top four markers with highest discrimination power showed no hits on BLAST2GO analysis.

Unexpected results were observed for two of the samples, *Arachis benthamii* (Handro) (PI 468162) and *Arachis correntina* (Burkart) Krapovickas & Gregory (PI 210554). The latter should have been placed by the analyses with the A genome (dark-blue group), but was co-located with the group of E genome; whereas *A. benthamii* which should have been with the E genome (pink group), was instead grouped with the A genome. Marker fingerprinting of a second sample from the same seed shipment of seed labeled as *A. correntina* (PI 210554) gave the same results. A third fingerprinting of seed labeled as *A. correntina* (PI 210554) performed using seeds from a second shipment from the germplasm collection eight months later and followed by new analyses by NJ, PCA and 3D-PCoA, showed results similar to the first two fingerprinting. The results for new seed of *A. benthamii* obtained from the bank of germplasm were also similar to the first fingerprinting. Photographs taken from two plants labeled as *A. correntina* (PI 210554) growing at the germplasm collection in Griffin, showed semi-erect or erect stems, had long leaflets and long rachis similar to the appearance of plants in section Erectoides, and not typical characteristics of the species *A. correntina*. *A. benthamii* was not currently being grown in the germplasm collection.

**Fig. 4:**
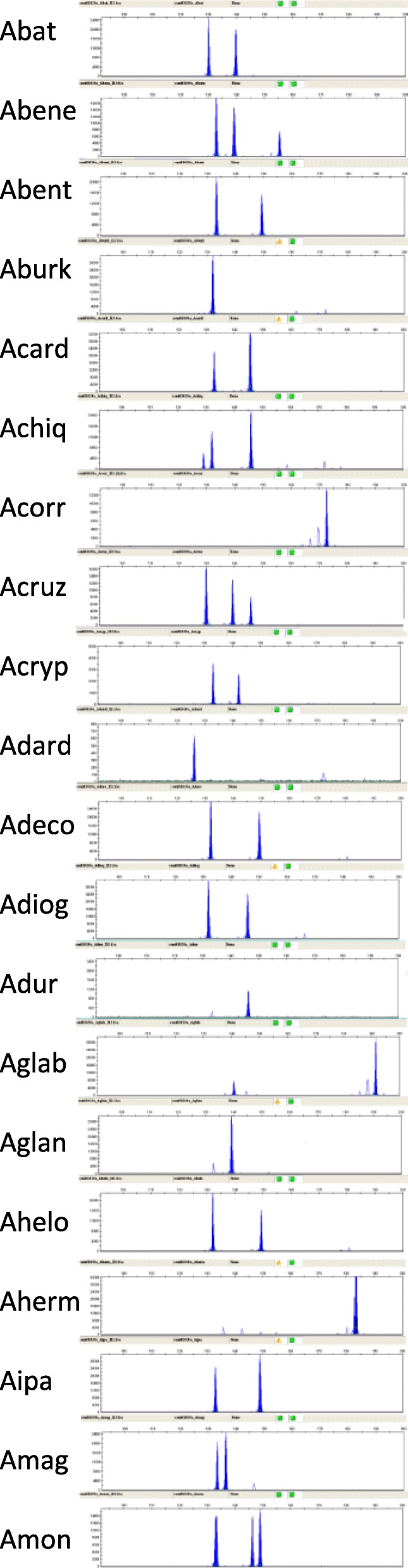
Electropherogram of amplicons generated on 20 wild peanut species using marker NPRL_cont01020a, this primer had UPIC score 16, that means 16 allele patterns were observed. X axis is in base pairs, and Y axis is fluorescence level. For graphic clarity the species names were shortened, please see List of Abbreviations

**Fig. 5:**
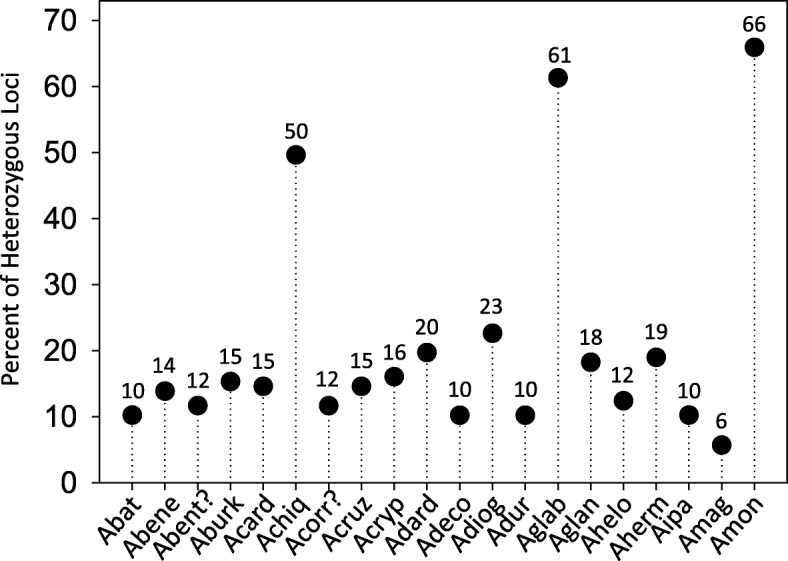
Percentage of heterozygous loci observed on 20 wild peanut species using 134 molecular markers that were transferable to all the species. For graphic clarity the species names were shortened, please see List of Abbreviations

One of the goals of this work was to provide molecular tools that can help identify germplasm in the peanut collection, and that can be used for accurate identification of each accession when screened for agronomic traits, *e.g*., resistance to aflatoxin accumulation. The initial list of 134 good quality markers was further condensed to keep only those with high levels of fluorescence and minimum or no background amplification, in addition to being polymorphic. This new set was comprised of 88 markers, including 71 from the present work and 17 from the literature. For these 88 markers we ran the script UPIC that provides the number of samples discriminated by each marker (UPIC score) and the combination of the minimum number of markers necessary to identify each of the samples tested. The UPIC scores for the best 88 markers are listed in Table 2; and the discrimination of all 20 accessions of wild peanut was accomplished by a combination of three new SSR markers: NPRL_cont01020a, NPRL_cont00528b and NPRL_BVZOG. Electropherogram results for marker NPRL_cont01020a (UPIC score: 16) are shown in Fig. 4. Among the 88 markers, 45 had DNA sequences with significant hits in BLAST2GO, these were related to: Transcription factors (5); Growth regulation (4); Signal Transduction (4); Sugar metabolism (4); Transport (4); Apoptosis (2); ATP receptor/synthesis (2); Embryogenesis (2); Organelle coat protein/receptor (2); Chloroplast synthesis (1); Glycosylation (1); Metabolic enzymes (1); Metal tolerance (1); Oligo transporter (1); Pentatricopeptide (1); Stress response (1); Translation (1); results are shown in Additional file 1: Table S1. The UPIC script was used to calculate heterozygosity of the samples at 134 loci, using the fingerprinting results of markers that amplified all the samples. Presence of two or more amplicons in a sample at one locus was considered heterozygous. The highest levels of heterozygosity observed were 66 %, 61 % and 50 %, and corresponded to *Arachis monticola* Krapovickas & Rigoni, *Arachis glabrata* Benth. (Harms ex Kuntze) Herm and *Arachis chiquitana* Krapovickas, Gregory & Simpson; the first two are tetraploids while the third one is a diploid. The lowest heterozygosity was observed in *Arachis magna* Krapov. *et al*. with only 6 % of heterozygous loci, Fig. 5. The heterozygosity level for the rest of the samples ranged from 10 % to 23 %.

**Table 2:** List of polymorphic and high quality markers (SSRs and InDels) screened with UPIC scripts. UPIC scores are the number of species with unique allele patterns out of 20 species tested

Marker	UPIC score	Marker	UPIC score	Marker	UPIC score	Marker	UPIC score	Marker	UPIC score
NPRL_cont01020a	16	Indel-003	7	NPRL_cont00952a	5	RM15C11	4	NPRL_cont00474a	2
NPRL_cont00528b	12	NPRL_cont00098a	6	NPRL_cont01065a	5	RN2F12	4	NPRL_cont00521a	2
Indel-016	10	NPRL_cont00393a	6	NPRL_ABCLW	4	RN34G06	4	NPRL_cont00816a	2
NPRL_cont00843a	10	NPRL_cont00659a	6	NPRL_cont00578a	4	AS1RN32E12	4	RN2H11	2
NPRL_cont00405a	9	NPRL_cont01572a	6	NPRL_cont00274a	4	NPRL_cont00179a	4	RN36A01	2
NPRL_cont00874a	9	NPRL_cont01622a	6	Ah-229	4	NPRL_cont00401a	4	AS1RN3E10	2
NPRL_cont00994a	9	NPRL_cont01294a	6	NPRL_cont00544a	4	RN3E10	4	NPRL_A5DQD	1
NPRL_cont00736a	9	NPRL_BVJSE	5	NPRL_cont00596a	4	NPRL_cont00346a	3	NPRL_cont00235a	1
AS1RI1F06	9	NPRL_BVZOG	5	NPRL_cont00741a	4	NPRL_cont00629b	3	NPRL_cont00479a	1
NPRL_cont00201a	8	NPRL_cont00151a	5	NPRL_cont00834a	4	NPRL_cont00686a	3	NPRL_cont00971a	1
NPRL_cont00793a	8	NPRL_cont00630a	5	NPRL_cont00981a	4	NPRL_cont00841a	3	NPRL_cont01170a	1
NPRL_cont01183a	8	NPRL_cont00658a	5	NPRL_cont01029a	4	NPRL_cont01080a	3	NPRL_cont01321a	1
NPRL_cont00626a	7	NPRL_cont01145a	5	NPRL_cont01663a	4	RN2C06	3	NPRL_cont01454a	1
NPRL_cont00629a	7	NPRL_cont01409a	5	NPRL_cont01893b	4	RN3B12	3	NPRL_cont01787a	1
NPRL_cont01077a	7	NPRL_cont01709a	5	NPRL_cont02088a	4	NPRL_cont00983a	3	NPRL_cont01984a	0
NPRL_cont01310a	7	NPRL_cont02651a	5	NPRL_cont02318a	4	NPRL_cont01604a	3	Indel-032	0
NPRL_AAJZM	7	RM14E11	5	NPRL_cont02432a	4	NPRL_cont00150a	2		
NPRL_cont01357a	7	NPRL_cont00461a	5	RM11H06	4	NPRL_cont00183a	2

**Fig. 6:**
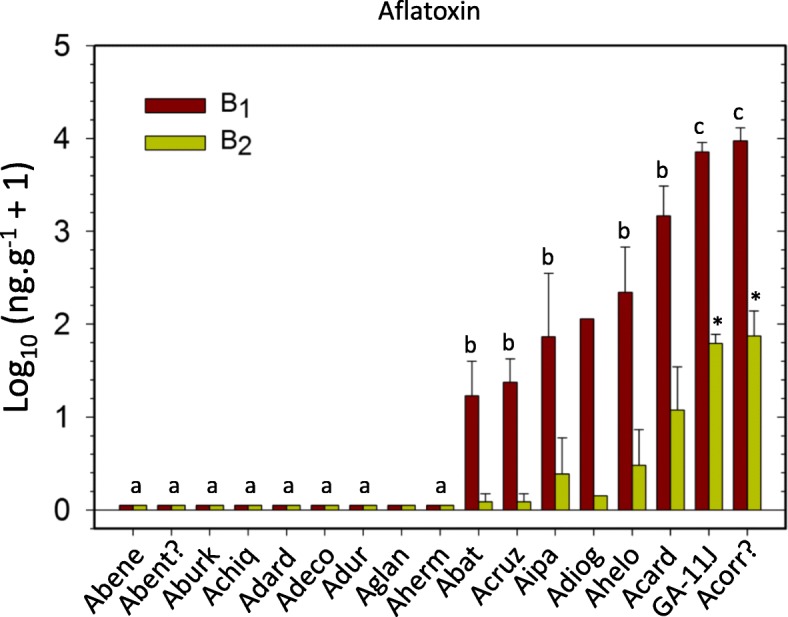
Concentration of aflatoxin B_1_ and B_2_ detected on individual seeds of 16 wild peanut species. Only 16, out of 20 species tested shown viability in *in vitro* culture, therefore were used in the analysis. Same letters indicate samples not significantly different from each other; samples without letters were not included in the statistical analysis. For graphic clarity the species names were shortened, please see List of Abbreviations

A second goal of this work was to screen the 20 wild species of peanut for aflatoxin accumulation using a method developed at the NPRL [27]. This method consists of challenging surface-sterilized half seeds with the application of spores of *Aspergillus flavus* NRRL 3357, followed by incubation and subsequent analysis of aflatoxins B_1_ and B_2_ using UPLC. No correlation was observed between the mass of *Aspergillus* mycelium or spores and the concentration of aflatoxins in the seeds. The small sample size used in this method, half cotyledon, was thought suitable for the limited supply of seeds available from the germplasm collections. Given the small size of the seeds for the present work, several modifications were made to the original protocol, and are described in the methods section. A critical new addition to the method was the test of viability through germination (in test tube) of each seed being challenged with the aflatoxigenic fungus. Half seeds containing the embryonic axis and placed in test tubes with Hoagland’s medium, were considered viable if the radicle emerged from the seed. Aflatoxin content quantified only from challenged “half seeds” that had a corresponding viable half were reported here. Only 16 of the 20 wild species of peanut showed viability, nine of them had no detectable levels of aflatoxins B_1_ and B_2_, the other seven wild species and the control (Georgia-G11J) showed accumulation of aflatoxin B_1_ between 7 and 19351 ng.g^-1^ seed, and B_2_ between 0.25 and 155 ng.g^-1^ seed. Data were converted to Log_10_ (x+1), and their mean and standard errors plotted, Fig. 6. Mean comparisons of the transformed values of aflatoxin B_1_ by all combinations of paired T-tests showed three groups significantly different (p ≤ 0.05), Fig. 6. Similar comparisons performed for the content of aflatoxin B_2_ detected only two different groups (*p* ≤ 0.05). Both aflatoxins, B_1_ and B_2_ were highest on Georgia-11J and on the sample received from the collection as *A. correntina* (PI 210554). Neither, aflatoxin B_1_ nor B_2_, were detected in *Arachis benensis*, *A. benthamii*, *Arachis burkatii*, *Arachis chiquitana*, *Arachis dardani*, *Arachis decora*, *A. duranensis*, *Arachis glandulifera* and *Arachis hermannii*, Fig. 6.

## Discussion

Improved tools are presented here, for the systematic screening of the small-size seeds of wild peanut species from germplasm collections to search for aflatoxin resistance; this approach uses single seed analysis, takes into consideration the seed viability and keeps record of the genetic fingerprinting of each accession. The complex genetics of aflatoxin resistance in peanut [24], combined with the large sample size required to reduce the variability of aflatoxin contamination data obtained in field experiments [26], have hindered the search of aflatoxin resistance as a trait. A significant reduction in sample size, and successful screening had been achieved using only 5 g of seeds per replicate, inoculated with *Aspergillus flavus* to detect aflatoxin resistance [6]. However, for the small size of wild peanut seeds, 5 g (in shell) would represent 36 seeds of *A. batizocoi* and 45 seeds of *A. dardani*, two species used in the present study; these are still very large numbers considering their limited supply. Thus, using single seeds per replicate as proposed here is a more suitable approach, and it allowed for statistical comparisons. Seed viability can be compromised by long term storage of peanut in banks of germplasm [53]. Thus, crucial to the protocol, is the testing of viability, which validates aflatoxin quantification as a result of the interaction between a live seed and the fungal pathogen. The results in Fig. 6, showed nine species with aflatoxin B_1_ and B_2_ below the minimum detection limit of the assay (0.10 ng.g^-1^ B_1_; and 0.01 ng.g^-1^ for aflatoxin B_2_). Overall, individuals with genomes A, B and K showed more accumulation of aflatoxins than those with genomes R1, PR, H and F. Though this study does not claim that the eight peanut species showing aflatoxin accumulation are susceptible, there is a higher probability of low or no resistance in accessions that accumulated aflatoxin; the experiment could be repeated to confirm potential candidates before their use in breeding programs. The same accessions of *A. duranensis* and *A. cardenasii* that here showed significant differences in aflatoxin accumulation had been reported with overall low levels of aflatoxin accumulation by Xue et al. 2004 in *in vitro* assays [6]. In that work, *A. cardenasii* had only 16-32 % the level of aflatoxin observed in *A. hypogaea* controls, and *A. duranensis* had between 12-24 % the level found in the controls. In our study *A. cardenasii* had 30 % of the level of aflatoxin in the control and *A. duranensis* had 0 %. To account for potential genetic variations within accessions of wild peanut species, we believe the genetic fingerprinting will help explain this type of variation.

In the present work, cluster analysis by Neighbor-Joining, PCA and 3D-PCoA were done for the purpose of visualizing the potential use of the markers in finding association between accessions according to their genomes described in the literature, and to keep record of the genetic fingerprinting of the samples. Markers NPRL_cont00626a, NPRL_cont01787a, NPRL_00179a and NPRL_cont00098a, discriminated 6, 4, 4, and 3 genomes, respectively; a total of 98 genome-discriminating markers are provided in Additional file 4: Table S4. In a study using 67 SSRs, Moretzsohn *et al*. (2004) found that accessions containing the same genome type tend to group together [43]; similar grouping according to genomes was also observed when intron sequences of single-copy genes were used to create a phylogenetic tree of multiple peanut species and accessions [54]. Given the high transferability across species for the markers reported in the present work, the high support by bootstrap (5000 replicates) observed on the dendrogram clades, Fig. 2, and the distinct groups observed in PCA and 3D-PCoA analyses, the grouping of the samples by genome seems to be clear. However, two samples were apparently misplaced; these were *A. benthamii* (PI 468162) that grouped with section Arachis group, and *A. correntina* (PI 210554) was placed with section Erectoides group. The PI 210554 has been extensively studied for decades, given its characteristic multiple disease resistance, and it was sometimes reported with different identification, for example as *A. villosa* ICG 8144 [55], *A. villosa* (PI 210554) [56], *A. villosa* (PI 210554) [57], *A. villosa* ICG 8144 [58], and as *Arachis* accession PI 210554 [59]. This prompted us to repeat the 373-marker fingerprinting of additional samples of PI 210554 received from the germplasm collection on different dates; and each time we obtained the same results, grouping PI 210554 with Erectoides samples instead of section Arachis group. It is possible, that at some point in time an error may have happened in the labeling of these two samples within the collection. The morphology of the plant growing at the germplasm collection was in concordance with the results of multivariate analysis of genetic fingerprinting, grouping it with Erectoides. The process of maintaining viable plant material of thousands of accessions is labor intensive and errors may occur; for example in the germplasm bank of avocado, up to 7% of loss identification has been reported [60]. The particularly high discrimination of the markers from the present work could be implemented as a resource for a cost-effective screening of the peanut germplasm collection. The placement of *A. burkatii* (2x) far from *A. glabrata* (4x) (section Rhizomatosae) is in accordance with the recent cytogenetic report by Ortiz et al (2017)[61] and ITS based phylogenetic analysis [62].

BLAST2GO analysis showed significant hits on 45 of the 88 best markers listed in Table 2. Polymorphism of SSR or InDel markers that showed hits on BLAST2GO, could result in amino acid changes within those proteins and therefore different phenotypes. Thus, the 45 markers with hits on transcription factors, stress response, signal transduction and growth regulation, could be valuable information for pre-breeding programs. We applied UPIC scripts, which is a decision tool for the cost-effective planning of experiments using fingerprinting [52], Table 2, and identified three markers which combined were able to discriminate all 20 wild peanut species. One of these three was NPRL_cont01020a, shown in Fig. 4, which had homology to a pentatricopeptide repeat-containing mitochondrial protein, such RNA-binding proteins regulate gene expression at the post-transcriptional level through RNA processing, splicing, stability, editing and translation, reviewed by Manna (2015) [63]. A second marker, NPRL_cont_00528b, had homology to another RNA-binding protein, the La-related protein LARP-1B, involved in the M-phase of the cell cycle in *Arachis duranensis*; LARP-1B homologs may function generally to control the expression of key developmental regulators in *Caenorhabditis elegans* [64], and in eukaryotes in general, binding transcripts of RNA Polymerase III [65]. The third marker in the group was NPRL_BVZOG, which in BLAST2GO showed homology to UDP-D-apiose/UDP-D-xylose synthase 1-like; mutations in this enzyme reduce apiose synthesis, thus preventing the cross-linking of rhamnogalacturonan II, and therefore the integrity of the plant cell wall [66]. Since the plant cell wall is the first barrier of protection against pathogens [67], the polymorphism of marker NPRL_BVZOG would be worth of further consideration. Overall, 92 % of the markers reported here, had only two hits or less (E 10^-5^) per A or B genome (*A. duranensis* and *A. ipaënsis*). The few markers that showed more than two hits per genome, or high copy number, are highlighted in yellow in Additional file 1: Table S1.

Heterozygosity shown in Fig. 5, matches the trends expected by ploidy level, for example, *A. monticola* is 2n: 40 [20, 68], and had 66%; *A. glabrata* the only tetraploid species known outside section Arachis, has 2n: 40 [69, 70] and presented a 61% heterozygosity. *A. chiquitana* was the only species that showed higher heterozygosity (50%) than expected. This species is listed as 2n=2x=20; however, it has a satellite greater than the sum of arm 1 and arm 2 [71]. This may explain the higher level of heterozygosity observed for this species. Phylogenetic anomalies have been observed on the SSR fingerprinting of three *A. chiquitana* accessions, where one accession clustered together with *A. diogoi* and another accession was distant from the rest [72]. This emphasizes the need of keeping a genetic fingerprinting record of accessions being tested.

## Conclusions

A set of cost-effective tools was developed to screen the small-size wild peanut seeds for aflatoxins. The method uses single seed analysis, considering potential loss of viability in storage, and keeping record of the genetic fingerprinting of each accession. A large new set of microsatellites and a combination of highly informative markers for screening are reported.

## Additional files

Additional file 1: **TableS1.** BLAST results for 345 DNA sequences from which the markers were designed, using the *Arachis duranensis* and *Arachis ipaënsis* genomes as databases. (XLSX 106 kb)

Additional file 2: **Table S2.** Primer sequences of the 288 SSRs developed in the present work. Number of alleles detected per marker, range of amplicon size, and BLAST2GO results for the sequences are also included. (XLSX 29 kb)

Additional file 3: **Table S3.** List of markers, InDels and SSRs used from the literature. (XLSX 34 kb)

Additional file 4: **Table S4.** Discrimination of genomes (*e.g*., H, D, F, AB, E, R1, R2) or groups of genomes (*e.g*., E+R2, B+F, D+H) by 98 markers organized by their quality (from excellent to doable). Genomes that were discriminated by a marker as group, were indicated with a “+” sign in between, *e.g*., F+D. Some cell colors correspond to the ones used in Fig. 2 and 3. (XLSX 32 kb)

## Abbreviations

Abat: Arachis batizocoi
Abene: Arachis benensis
Abent: Arachis benthamii
Aburk: Arachis burkatii
Acard: Arachis cardenasii
Achiq: Arachis chiquitana
Acorr?: Arachis correntina?
Acruz: Arachis cruziana
Acryp: Arachis cryptopotamica
Adard: Arachis dardani
Adeco: Arachis decora
Adiog: Arachis diogoi
Adur: Arachis duranensis
Aglab: Arachis glabrata
Aglan: Arachis glandulifera
Ahelo: Arachis helodes
Aherm: Arachis hermanii
Aipa: Arachis ipaënsis
Amag: Arachis magna
Amont: Arachis monticola
3D-PCoA: 3-D Principal Coordinate Analysis
BLAST: Basic Local Alignment Search Tool
BLAST2GO: BLAST to Gene Ontology
InDel: Insertion Deletion
IVSC: In Vitro seed colonization
NJ: Neighbor Joining
NPRL: National Peanut Research Laboratory
PCA: Principal Component Analysis
PGRCU: Pl. Genetic Res. Conservation Unit
SSR: Simple sequence repeat
UPIC: Unique Pattern Informative Combinations
UPLC: Ultra-performance liquid chromatography
